# In Vitro and In Vivo Antidiabetic, α-Glucosidase Inhibition and Antibacterial Activities of Three Brown Algae, *Polycladia myrica*, *Padina antillarum*, and *Sargassum boveanum*, and a Red Alga, *Palisada perforata* from the Persian Gulf

**DOI:** 10.5812/ijpr-133731

**Published:** 2023-04-17

**Authors:** Niloofar Moheimanian, Hossein Mirkhani, Azar Purkhosrow, Jelveh Sohrabipour, Amir Reza Jassbi

**Affiliations:** 1 Medicinal and Natural Products Chemistry Research Center, Shiraz University of Medical Sciences, Shiraz, Iran; 2 Department of Pharmacology, School of Medicine, Shiraz University of Medical Sciences, Shiraz, Iran; 3 Department of Natural Resources Researches, Agriculture and Natural Resources Research and Education Center, Agricultural Research, Education and Extension Organization (AREEO), Bandar Abbas, Iran

**Keywords:** Algae, Antibacterial, Blood Glucose Level, STZ-induced Diabetic Rats

## Abstract

**Background:**

In recent decades, algae have attracted worldwide attention for their great biological activities, such as antidiabetic and antibacterial properties.

**Objectives:**

We measured antibacterial and α-glucosidase inhibition potential of methanol and 80% methanol extracts of three brown algae species, *Polycladia myrica*, *Padina antillarum*, and *Sargassum boveanum*, and a red alga, *Palisada perforata*, from the Persian Gulf coasts.

**Methods:**

Antibacterial activity of the algal extracts was assessed by broth dilution method against three gram-negative and -positive bacteria, including *Escherichia coli*, *Klebsiella pneumonia*, *Pseudomonas aeruginosa*; *Staphylococcus epidermidis*, *Staphylococcus aureus*, and *Bacillus subtilis*, respectively. Furthermore, the yeast’s α-glucosidase inhibition of the algal extracts was measured via colorimetric assay. In addition, we investigated the beneficial effect of 80% MeOH extract of *S. boveanum* on the blood glucose levels in streptozotocin-induced diabetic rats.

**Results:**

The MeOH extract of *S. boveanum* was the best antibacterial extract with MIC = 2.5 mg/mL against all bacterial strains except for *E. coli*. The MeOH and 80% MeOH extracts of *P. myrica* and *P. antillarum* inhibited α-glucosidase at most with IC_50_ values of 12.70 ± 1.88 µg/mL and 13.06 ± 4.44 µg/mL, respectively. The oral gavage of *S. boveanum* extract in streptozotocin- (STZ-) induced diabetic rats resulted in decreasing their postprandial blood glucose levels. The algae and acarbose decreased blood glucose levels after sucrose administration in 60 minutes, compared to the non-drug-treated animals, with p values of 0.03 and 0.007, respectively.

**Conclusions:**

Overall, due to the in vitro and in vivo antidiabetic potential of *S. boveanum*, we suggest the alga as a new source for the isolation and identification of potential antidiabetic and antibacterial compounds.

## 1. Background

Nowadays, red and brown macro-algae get worldwide attention to stimulate many investigations on different biological activities, for instance, antioxidant and antiproliferative (1), anticoagulant (2), antitrichomonal (3), antileishmanial (4), antibacterial (5, 6) and antidiabetes (7). Developing new antidiabetic agents is vital for managing diabetes mellitus, especially Type 2 (T2DM), which includes 90% of the world's diabetic population (8). Different researchers have been interested in exploring the antidiabetic potential of marine algae since they contain various antidiabetic metabolites, including polyphenolics, polyunsaturated fatty acids, and dietary fibers (9). Daily consumption of algae in diabetes diet can reduce blood glucose concentration after meals (10, 11). In this research, we explore the α-glucosidase enzyme inhibition potential of the algae because of the reasonable price, availability, and simplicity of the bioassay. In addition, inhibiting the enzyme overwhelms the boost of high blood-sugar levels after taking carbohydrate diets (12, 13). Acarbose and voglibose are the two standard α-glucosidase inhibitors that successfully decline postprandial glucose levels and are prescribed for managing T2DM (14). Due to the side effects of these medicines, such as diarrhea, bloating, and abdominal pains, numerous investigations have been done to find new enzyme inhibitors with fewer side effects (15).

The Persian Gulf is one of the harshest ecosystems in the world because of its high temperature, salinity, and sharp sunlight (16, 17). These key factors qualify it to host some of the most magnificent marine fauna and flora, including brown and red algae. Over 150 species of marine algae are reported from the Persian Gulf (18, 19). Biological activities and chemical contents of some algae collected from the Persian Gulf coastlines have been determined recently. For instance, antioxidant activity and total phenolic contents of various fractions of *Sargassum swartzii*, *Polycladia myrica*, and *Colpomenia sinuosa* were studied, which showed the superiority of *S. swartzii* (20). In addition, ethyl acetate and MeOH extracts of some marine algae, *Ulva flexuosa* and two genera of *Padina*, showed great antimicrobial and cytotoxic activities (21). Anti-melanogenesis activities of 17 species of 4 brown algae genera, *Padina*, *Colpomonia*, *Cystoseira*, and *Sargassum*, from the Northern Coasts of the Persian Gulf, were investigated, among which *Padina boergesinii* showed the highest activity (22). In addition, different liquid-liquid extracted fractions of *Padina australis* showed cytotoxic activity against cancerous cell lines (23). The hexane-soluble parts of methanol-ethyl acetate extract of *Sargassum plagyophylum* showed antidepressant effects in a mice model of despair (24). The red and brown algae from the Persian Gulf, *Gracilaria corticata*, *Gracillaria salicornia*, and *Sargassum oligocystum* exhibited strong antileishmanial activities (25).

Chemical analysis of various marine organisms resulted in the isolation of more than 1400 new compounds in 2020 (26), while based on the online database: MarinLit (https://marinlit.rsc.org/) until now (7th February 2023), 39807 compounds have been identified in marine organisms. Among them, diverse classes of antidiabetic compounds were characterized in algae species using in vitro and in vivo bioassay-guided studies (9, 27, 28). For instance, fucoxanthin, a marine carotenoid, is one of the brown algae's most effective nutraceutical compounds with great antibacterial and antidiabetic characteristics (29, 30). In addition to fucoxanthin, several α-glucosidase inhibitors belonging to terpenoids, phenolics, and echols have been extracted from different algae (31-33). Two α-glucosidase inhibitors with bromobenzene structures were isolated from a brown alga; *Dictyopterishoytii* (34), while a potent enzyme inhibitor; ishophloroglucin A that is a novel phlorotannin, was isolated from a brown alga, *Ishige Okamura* (Yendo) (35). Recently, two new brominated metabolites, ethyl methyl-2-bromobenzene 1,4-dioate and diethyl-2-bromobenzene 1,4-dioate, from a brown alga *Dictyopteris hoyti*, in the coastal region of Raysut, Oman, showed great α-glucosidase inhibitory activity (34). Polyphenolic fraction of a red alga *Symphyocladia latiuscula* showed antidiabetic effect in diabetic rats by improving the electrophysiological parameters (36). Eckol is a phlorotannin extracted from *Ecklonia* species, which showed potent antidiabetic activity (37).

In our previous work, α-glucosidase activity of MeOH and 80% MeOH extracts of *C. sinuosa*, *Sargassum* acinaciforme, Iyengaria stellata, *Sirophysalis trinodis* and two accessions of *P. myrica*, from the Persian Gulf coasts were examined. Furthermore, we studied the chemical constituents and the effects of 80% MeOH extract of *S. trinodis* on blood glucose in STZ-induced diabetic mice (38).

## 2. Objectives

In this project, we study antibacterial and α-glucosidase inhibitory activities of three brown algae species, *P. myrica*, *Padina antillarum* and *Sargassum boveanum*, and a red alga, *Palisada perforata*, collecting from the intertidal regions of the Persian Gulf coastlines. Then, we chose *S. boveanum* for further in vivo assay due to its better α-glucosidase inhibition potential and enough amount of the extract required for the in vivo test.

## 3. Methods

This section is embedded in a supplementary file including the following items: Reagents; collection and extraction of the algae (Appendix 1 and 2); thin layer chromatography (TLC) analytical conditions (Appendix 3); antibacterial bioassay using nutrient broth microdilution method; kinetic of inhibition patterns on α-glucosidase (Appendix 4); experimental animals: Induction of diabetes; statistical analysis.

## 4. Results

The algal botanical characterizations of the algae are presented in Appendix 1, while the algal extraction parameters, including solvent types, volume, and extraction yield, are given in Appendix 2. The minimum inhibitory concentration (MIC) of the MeOH and 80% MeOH extracts of the algae against the tested bacterial growth are reported in Table 1. The MeOH extract of *S. boveanum* was the most active antibacterial extract. All tested microorganisms were susceptible to it at MIC values of 2.5 mg/mL except that *E. coli*. In addition, 80% MeOH extracts of *P. myrica* and *S. boveanum* showed the strongest activity against the growth of *E. coli* at MIC 0.62 mg/mL. Furthermore, the MeOH extract of *P. myrica* had moderate antibacterial activity against the growth of all bacteria but not those of *P. aeruginosa* and *K. pneumoniae*. On the other hand, the MeOH and 80% MeOH extracts of *P. perforata* and *P. antillarum* exhibited the weakest result, with no activity against any bacteria in the antimicrobial bioassay.

**Table 1.** Antibacterial Potential of the Algal Extracts Expressed as MIC (mg/mL), Using Nutrient Broth-micro Dilution Method

Microorganisms	Algae (Rows)					
	*S.a.*	*S.e.*	*B.s.*	*P.a.*	*E.c.*	*K.p.*
***Sargassum boveanum*** ** (MeOH)**	2.5	2.5	2.5	2.5	NA	2.5
***Sargassum boveanum *** ** (80% MeOH)**	2.5	NA	NA	NA	0.62	NA
***Palisada perforata*** ** (MeOH)**	NA	NA	NA	NA	NA	NA
***Palisada perforata*** ** (80% MeOH)**	NA	NA	1.25	NA	2.5	NA
***Padina antillarum*** ** (MeOH)**	NA	NA	2.5	NA	1.25	NA
***Padina antillarum*** ** (80% MeOH)**	NA	NA	NA	NA	NA	NA
***Polycladia myrica*** ** (MeOH)**	2.5	2.5	2.5	NA	2.5	NA
***Polycladia myrica*** ** (80% MeOH)**	NA	NA	2.5	2.5	0.62	NA
**Chloramphenicol**	0.0125	0.025	0.012	0.05	0.05	0.05

Abbreviations: NA, not active (> 5); S.a., *Staphylococcus aureus*; S.e., *Staphylococcus epidermidis*; B.s., *Bacillus subtilis*; P.a., *Pseudomonas aeruginosa*; E.c., *Escherichia coli*; K.p., *klebsiella pneumoniae*.

**Figure 1.**
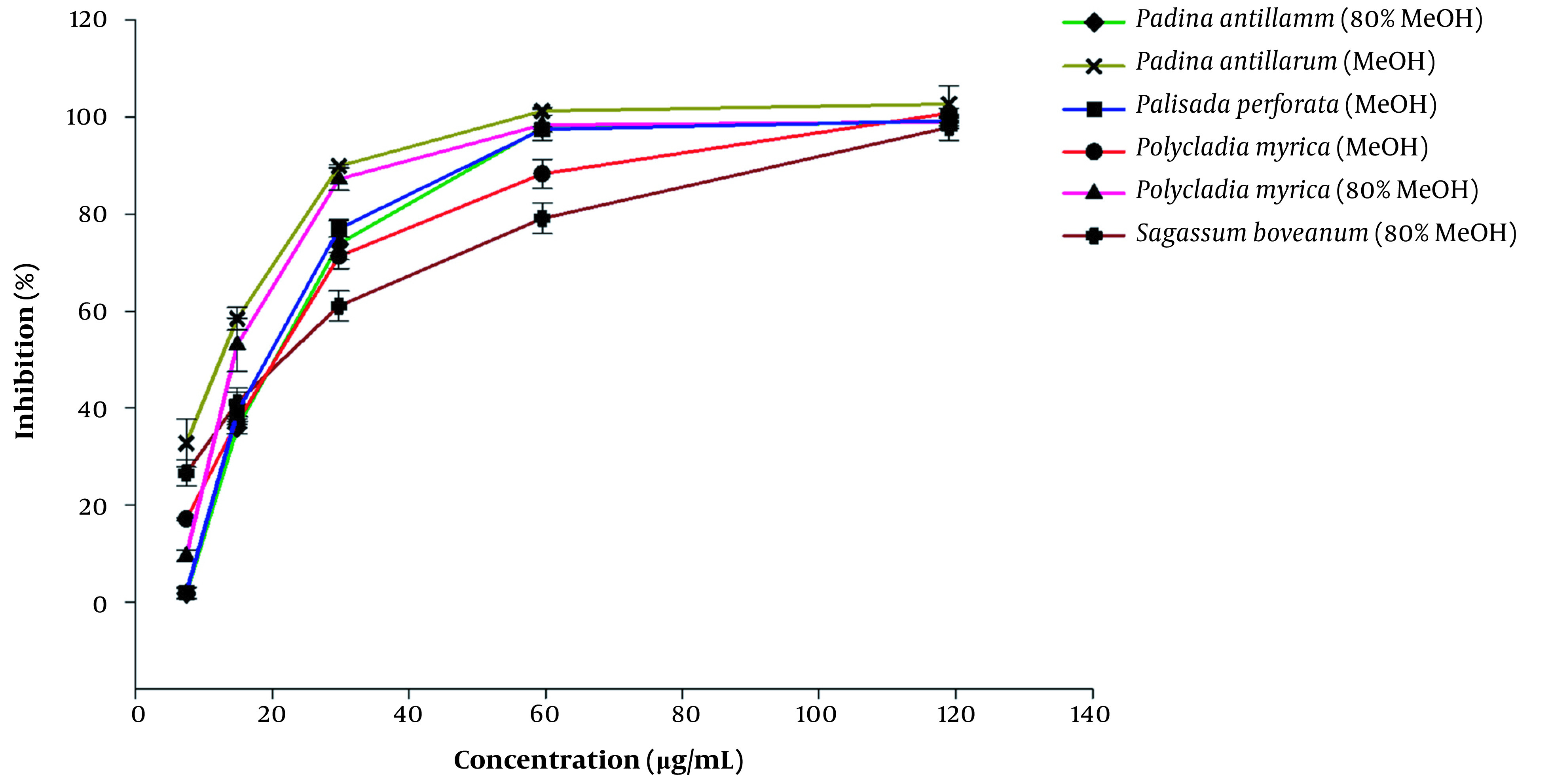
Inhibition percentages for the active algae extract at different concentrations (8, 15, 30, 60, and 120 µg/mL) against α-glucosidase. Acarbose was used as a standard drug with an IC_50_ value of 160.15 µg/mL (The curve is out of range). Results are presented as mean ± SE of three experiments (n = 3).

This is the first report on the α-glucosidase enzyme inhibitory activity of the above-mentioned algal extracts (Table 2). The smaller IC_50_ values indicate that lower concentrations of the algal extracts are utilized to inhibit half of the α-glucosidase enzyme activity. The algal extracts showed several-folds higher α-glucosidase inhibition potential with IC_50_ 12.7 - 21.17 µg/mL, compared to those measured for the standard drug; acarbose with IC_50_ value of 160.15 ± 27.52 µg/mL (P value < 0.05). The highest inhibition was achieved by 80% MeOH extract of *P. myrica* with the lowest IC_50_ value of 12.70 ± 1.88 µg/mL, while the 80% MeOH extract of *P. perforata* and MeOH extract of *S. boveanum* were inactive even at IC_50_ value of 1000 µg/mL. Furthermore, Figure 1 illustrates the dependency of the α-glucosidase inhibition percentage of the applied dosage of the algal extracts in the bioassay. The kinetics of the enzyme was carried out using the extracts of *S. boveanum* against α-glucosidase inhibition (Appendix 3).

**Table 2.** IC_50_ Values for Inhibition of α-glucosidase Test of the Algae Extracts and Acarbose ^a^

Algae	Enzyme Inhibition IC_50_ (µg/mL)
***Sargassum boveanum*** ** (MeOH)**	> 1000
***Sargassum boveanum*** ** (80% MeOH)**	19.66 ± 1.94
***Palisada perforata*** ** (MeOH)**	19.09 ± 1.80
***Palisada perforata*** ** (80% MeOH)**	> 1000
***Padina antillarum*** ** (MeOH)**	13.06 ± 4.44
***Padina antillarum*** ** (80% MeOH)**	21.17 ± 1.55
***Polycladia myrica*** ** (MeOH)**	20.97 ± 2.75
***Polycladia myrica*** ** (80% MeOH)**	12.70 ± 1.88
**Acarbose (standard)**	160.15 ± 27.52

^a^The IC_50_ values were calculated using at least five serially diluted solutions of each extract and by linear regression. These values are the means ± standard error of three replicates for each sample.

**Figure 2.**
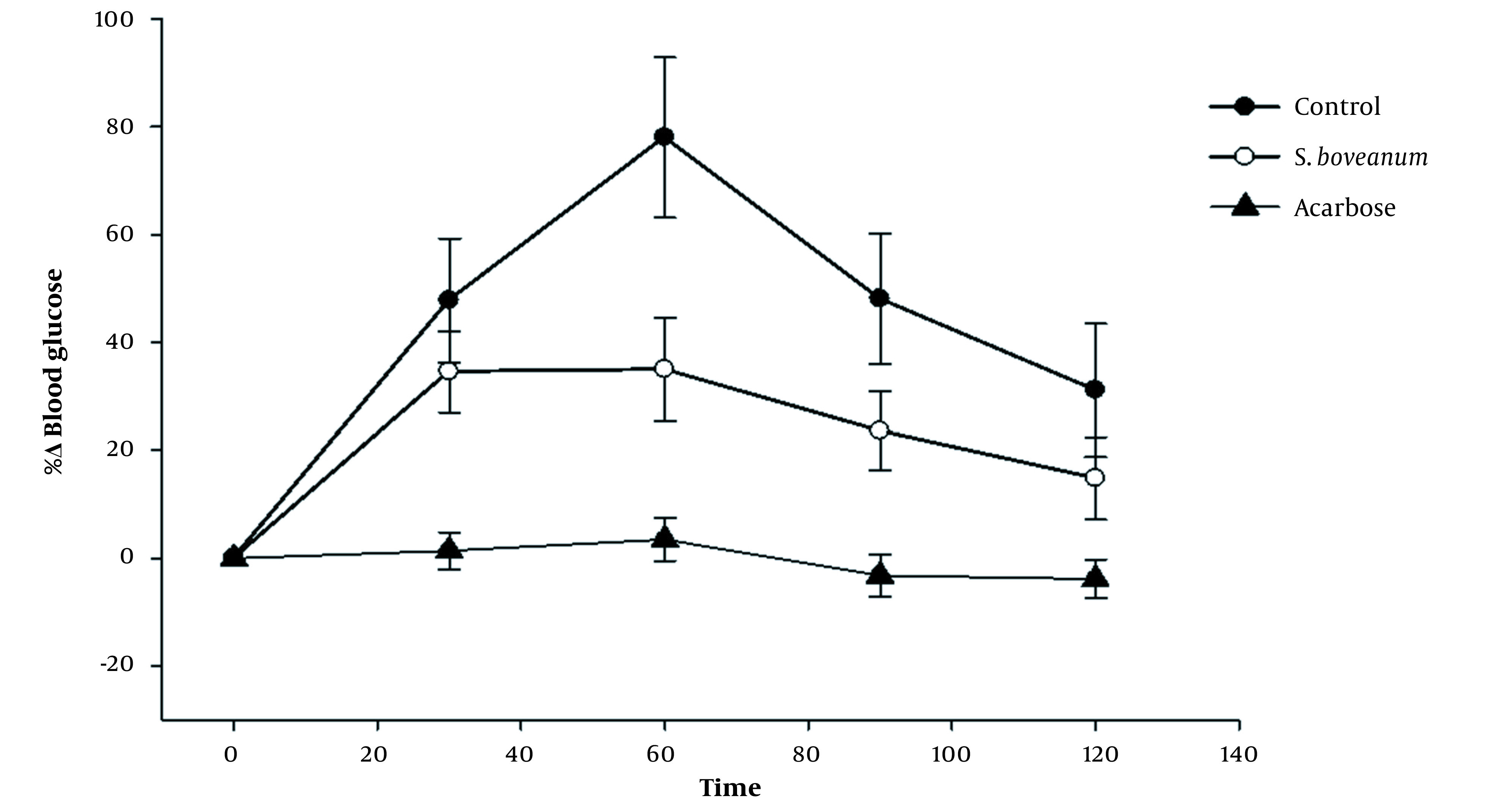
The percentage change (%Δ)^*^ of blood glucose levels in STZ-induced diabetic rats at different time points (0, 30, 60, 90, and 120) for three groups of diabetic animals. Control group (negative control), without any treatments other than sucrose. The acarbose group (as positive control) and *Sargassum boveanum* group (case group) received acarbose and the algal extract, respectively, 30 min before sucrose administration. Each value is expressed as mean ± SE. ^*^%Δ = [(BG)_n_ - (BG)_0_] × 100/ (BG)_0_, (BG)_n_ is blood glucose levels (mg/dL) in the times 30, 60, 90,120, and (BG)_0_ is blood glucose levels (mg/dL) in the time 0.

The 80% MeOH extract of *S. boveanum* was selected for the in vivo test. The effect of the algae on after-meal-blood glucose levels was measured in thirty diabetic rats induced by STZ. Postprandial blood glucose levels of the algae-administered rats were lower than those of the rats used as the control group (Figure 2). After taking sucrose, the rats' blood glucose levels rose by 78% at 60 min to their maximum levels. Furthermore, after administrating algal extract to STZ-induced diabetic rats, the rise in postprandial blood glucose levels was suppressed (P-value < 0.05) by 34.6, 35.0, 23.6, and 14.9 % at 30, 60, 90, and 120 min, respectively.

Moreover, the statistical outcomes demonstrated that there are no significant differences between the algae extract and acarbose in diminishing blood glucose levels 60 minutes after sucrose administration. On the other hand, the results between the blood glucose levels of diabetic controls and the other two groups, at the same time, are significantly different (Table 3). These results indicated that *S. boveanum* retards the absorption of dietary carbohydrates in the meal and, as a result, suppresses the rise in postprandial blood glucose levels.

**Table 3.** Statistical Comparison of the Blood Glucose Levels of the Studied Groups 60 Minutes After Sucrose Administration ^a,b,c,d^

Group 1	Group 2	Δ BG (Group 1 – Group 2)^e^	P Value	95% Confidence Interval	
				Lower Bound	Upper Bound
**Control (n = 9)**	Acarbose (n = 10)	192.97	0.007	47.15	338.78
***Sargassum boveanum*** ** (80% MeOH) (n = 11)**	Acarbose	38.03	0.84	- 100.63	176.69
***Sargassum boveanum*** ** (80% MeOH)**	Control	- 154.94	0.03	- 297.11	- 12.77

^a^ Values are the results of 9-11 experiments. Statistical analysis was carried out using one-way analysis of variance (ANOVA) followed by post-hoc Dunnett's test.

^b^ Control: Diabetic animals received sucrose (2 g/Kg)

^c^ Acarbose: Diabetic animals received acarbose (30 mg/Kg) 30 min before sucrose administration.

^d^* Sargassum boveanum*: Diabetic animals received algae extract (30 mg/Kg) 30 min before sucrose administration.

^e^ Difference of mean BG (blood glucose) level (mg/dL) between groups (Group 1- Group 2)

We have analyzed MeOH and 80% MeOH algal extracts using silica gel TLC as a fingerprinting chromatogram and a guide for the future isolations of the active constituents (Appendix 3).

## 5. Discussion

Previously, the antibacterial and antifungal properties, antioxidant activity, and total phenol contents of MeOH and aqueous-methanol extracts of *S. boveanum* collected from Qeshm Island were investigated by our research group (39). Unlike in the present study, the 80% MeOH extracts of *S. boveanum* in the earlier report did not show any activity against the growth of *E. coli*. In addition to the aqueous methanol extract, the MeOH extracts of *S. boveanum* in the current study exhibited wide-spectrum antibacterial activity against all evaluated microorganisms except *E. coli*. Overall, the difference between the antibacterial activities exhibited by one species in different studies may be due to the environmental conditions of their collection locations. Similarly, the collection's location of *P. myrica* accessions affected their enzyme inhibitory activities. Among them, the extracts of *P. myrica* collected from Ziarat, and Bandar Lengeh (Table 2), were more potent than those from Owli-ye-jonubi in Bushehr Province (38).

In addition to the species collected from the Persian Gulf, among some brown algae collected from the coast of Canakkale, Turkey, a MeOH extract of *Cystoseria compressa* was the most active one against different bacterial strains in vitro tests (40). Finally, an ethanol extract of *Sargassum polycystum* (From Tanjung Tuan, Port Dickson) exhibited weaker antimicrobial activity against *Klebsiella pneumonia* with MIC value of 6.25 mg/mL compared to that we report here for *S. boveanum* (2.5 mg/mL) (41). A methanol extract of *P. antillarum* collected from the coast of Karachi in Pakistan was used to treat diabetic mice. The algal extract reduced the body weight and decreased the levels of triglycerides, while elevating HDL cholesterol levels in the test animal's blood (42).

The lack of antibacterial activity for the MeOH and 80% MeOH extracts of *P. perforata* and *P. antillarum*, respectively, in the present study (Table 1) is due to the absence of active constituents in the collected species (43). On the other hand, the effect of extracting solvents (44), harvesting seasons (43), and degrading the active constituents during the drying process, for instance, the antioxidant flavonoids in *P. perforata* (45), may be other possible reasons for these effects. One of the reasons for the lower antibacterial potential observed for the algae compared to the positive controls is suggested to be due to their different chemical structures that could affect the in vitro bioassays, such as the various diffusion coefficients of different metabolites in the growth medium of the bacterial test. 

In our current study, the antidiabetic effects of some studied species have also been investigated. Among the tested algal extracts, both *P. antillarum* and *P. myrica* extracts inhibited enzyme activity of α-glucosidase several folds higher than that inhibited by acarbose (Table 2, P-value < 0.001). On the other hand, the 80% MeOH extract of *S. boveanum* inhibited α-glucosidase at lower concentrations compared to the control drug, while its MeOH extract exhibited no activity in the maximum tests concentration (> 1000 µg/mL, Table 2). The reverse effects of extracting solvents were observed for the alga, *P. perforata*. Our results show that the solvent composition has a key role in extracting bioactive compounds, which depend on different types of chemical constituents of various algae species. These effects are compatible with previous research on the α-glucosidase inhibition potential of water and ethanol extracts of some Irish brown algae (46). Among the tested algae, the water extract of *Alaria esculenta* inhibited the enzyme better than that of the ethanol extract of *Himanthalia elongata* (46).

Interestingly, the ethyl acetate extract of *S. boveanum* from the Bandar Abbas coastal area exhibited great α-amylase inhibitory activity (47). In that study, the MeOH extracts of *S. trinodis*, *P. perforata*, and *P. myrica* also showed high α-amylase inhibition (47). In addition, α-amylase potential of ethyl acetate extract of *P. myrica* (IC_50_ = 0.72 mg/mL) and MeOH extract of *P. perforata* (IC_50_ = 1.1 mg/mL) was close to that of their applied standard drug; acarbose (IC_50_ = 0.75 mg/mL) (47). However, since the α-glucosidase inhibitory activity of the last two algae, showed that the MeOH extract of *P. perforata* (IC_50_ = 19.09 ± 1.80 µg/mL) and MeOH and 80% MeOH extract of *P. myrica* (IC_50_ = 20.97 ± 2.75 and 12.70 ± 1.88 µg/mL, respectively) had better activity than acarbose (IC_50_ = 160.15 ± 27.52 µg/mL, p value < 0.05), we may consider them as good candidates for in vivo tests. Also, α-glucosidase inhibitory activity for ethanolic extracts of 19 Korean marine macroalgae species was investigated. Similar to our results, the best measured IC_50_ values of their inhibitory activity were 2.17 μg/mL and 101.62 μg/mL (48). In a survey of α-glucosidase activity of some macro-algae collected from Gulf of Izmir, three brown algae of the genus *Cystoseira*, including *C. barbata* (90.7%), *C. compressa* (89.8%) and *C. crinita* (91.9%), exhibited the comparable potential to that measured for acarbose (79.5%), when tested at 1 mg/mL (49). In addition, the antidiabetic activity of some Malaysian algae was investigated, which showed the highest inhibitory activity against α-glucosidase for water extracts of *Halimeda macroloba*, green algae species, with an IC_50_ value of 6.388 mg/mL (50). In another report, ethanolic extract of *Sargassum wightii* genus exhibited strong α-glucosidase inhibitory activity (IC_50_ = 6 mg/mL) (51). Our results showed the potency of our investigated algae compared to other reports. 

Data analysis of the kinetic of the enzyme revealed that the increase in the extract's concentration had not affected the Vmax and remained at about 0.09 mM/min. In contrast, Km was increased significantly by 3.0, 5.4, and 24.9 mM, respectively (Appendix 4). These results indicated that the extract inhibits α-glucosidase in a competitive manner. Moreover, in vivo tests have been done for 80% MeOH extract of *S. boveanum* to achieve more reliable data. STZ-induced diabetic rats had oral gavage with the algae after sucrose administration, which showed a rise in postprandial blood glucose level until 60 min, and then decreased to 14.8%. Similar to our results, in a group of diabetic mice administered by diphlorethohydroxycarmalol, a phlorotannin isolated from brown algae, glucose levels decreased compared to the control group (32). Heophorbide A with a tetrapyrrole structural moiety was isolated from red algae and showed the same blood glucose-decreasing effect in diabetic rats (52). The phlorotannins isolated from an Egyptian accession of *C. compressa* exhibited antidiabetic effects in streptozotocin-induced diabetic rats by improving serum insulin compared to the diabetic group (53).

In the TLC chromatogram of the algal extracts, after visualizing by 1% thymol reagent (Appendix 3) exhibited three major pink spots with Rf values between 0.13 and 0.46 were present in all extracts except *S. boveanum*, while two dark spots appeared with Rf values between 0.72 and 0.83 in the MeOH and 80% MeOH extracts of *S. boveanum* and MeOH extract of *P. perforata*. The pink spots suggest the glycoside character of the compounds (39). Due to the great results of investigated algae, especially *S. boveanum*, in the future, we will isolate secondary metabolites of active extracts responsible for the mentioned activities, which could be suggested for applications in medicinal and pharmaceutical purposes. 

### 5.1. Conclusions

In this research, we have investigated the antibacterial activity and the inhibitory effect of MeOH and 80% MeOH extracts of 4 different algae on the α-glucosidase enzyme. The MeOH extract of *S. boveanum* revealed the best results in antibacterial activity among all other algae extracts. Moreover, almost all of the extracts showed great α-glucosidase inhibitory activity, which makes them decent nominees for more advanced examinations. We choose one of them, *S. boveanum*, to evaluate its antidiabetic effect in vivo test as the first step to achieve this goal. The algae represented satisfactory results in decreasing glucose levels in diabetic rats compared to acarbose. Thus, we suggested *S. boveanum* as a good candidate for further investigations, such as isolating its α-glucosidase inhibitory active metabolites. In addition, we recommend the algae be tested as a nutraceutical for diabetes, which can also be studied in other therapeutic fields, such as weight control.

ijpr-22-1-133731-s001.pdf

## Data Availability

The data presented in this study are uploaded during submission as a supplementary file and are openly available for readers upon request.
