# Supplementary material for: In Vitro and In Vivo Antidiabetic, α-Glucosidase Inhibition and Antibacterial Activities of Three Brown Algae, Polycladia myrica, Padina antillarum, and Sargassum boveanum, and a Red Alga, Palisada perforata from the Persian Gulf
Source: Iran J Pharm Res. 2023 Apr 17;22(1):e133731. doi: 10.5812/ijpr-133731 (PMC10728852; doi:10.5812/ijpr-133731)
Supplement: ijpr-22-1-133731-s001.pdf [file ijpr-22-1-133731-s001.pdf]

In vitro and _in vivo_ antidiabetic, α-glucosidase inhibition and antibacterial activities of three brown algae, _Polycladia myrica_, _Padina antillarum_ and _Sargassum boveanum_, and a red alga, _Palisada perforata_ from the Persian Gulf

Experimental

Reagents

α-Glucosidase (from yeast, EC 3.2.1.20), *p*-nitrophenyl-α-D-glucopyranoside (PNPG) and acarbose were obtained from Sigma-Aldrich Company (Germany) while all the solvents were prepared from Merck Chemical Company. Chloramphenicol, 3-(4, 5-dimethylthiazol-2-yl) -2, 5-diphenyltetrazolium bromide (MTT) and streptozotocin were purchased from Sigma-Aldrich (Germany) and *P*-iodonitrotetrazolium violet (INT) was obtained from Fluka. All of the reagents used for this study were of analytical grade.

Collection and extraction of the algae

All algae samples were collected from various locations on the coastlines of Iran (Persian Gulf) in the depth of about -1.0 m in March 2015 (Appendix 1). All samples were identified by J. S., the algal taxonomist of Agriculture and Natural Resources Research and Education Center of Hormozgan Province, Iran. At least one voucher sample is kept in the herbarium of the Medicinal and Natural Products Chemistry Research Center, Shiraz University of Medical Sciences and a voucher number is given to each individual alga (Appendix 1).

After collection of the algae, each fresh sample was separately cleaned, washed, chopped, weighed and then extracted consecutively in certain volumes of 80% MeOH and pure MeOH by maceration method at room temperature (Appendix 2, 2 × 24h). Afterwards, each filtered extract was evaporated under low pressure at 40 ºC to afford crude extracts of the above-mentioned

solvents (Appendix 2). For bioassays, the dried extracts of each sample was prepared by dissolving in the extracting solvents to achieve desired concentrations.

Thin layer chromatography (TLC) analytical conditions

We analysed the chemical constituents of the extracts using pre-coated TLC plates (silica gel 60 F254, 0.25 mm film thickness, Merck). To achieve ideal resolution and retention factor (R_f) various mobile phase composition were evaluated. The silica gel TLC analysis was performed using ethyl acetate: MeOH: formic acid (7:3.5:1) as the mobile phase followed by the spray with 0.5% thymol-sulfuric acid solution in 95 mL EtOH and heating at 100 $^{\circ}$C which resulted in detection of pink-coloured which indicated the presence of glycosides (Appendix 3) (1).

Antibacterial bioassay using nutrient broth microdilution method

For antibacterial activity evaluation of the algal extracts, nutrient broth micro-dilution (NBMD) method was selected to calculate the minimum inhibitory concentrations (MICs) based on the previously described method (2). This assay was performed against three Gram-negative bacteria (*Escherichia coli*: PTCC1330, *Klebsiella pneumoniae*: PTCC1053, and *Pseudomonas aeruginosa*: PTCC 1074, and three Gram-positive bacteria (*Staphylococcus aureus*: PTCC1112, *Staphylococcus epidermidis*: PTCC1114, *Bacillus subtilis*: PTCC1023).

Serial dilutions of the algal extracts or chloramphenicol as the positive control were dissolved in dimethyl sulfoxide (DMSO) in desired concentrations. Subsequently, 95 µL of the nutrient broth and 5 µL of the samples were added into a 96-well microplate and then mixed with 100 µL of bacterial suspension (OD = 0.1 at 600 nm) and incubated for 24 hours at 37 ºC. Then 0.5% INT solution in water was prepared and 10 µL of the solution was added to each well in

microplate. Afterwards, the microplates were incubated for further 30 min, at the above-mentioned condition. At the end, MICs of the extracts or antibacterial standard were defined as the lowest concentration that discoloured the purple solution of INT. The discolouration indicated the inhibition effects of the algal extracts on the tested bacterial strain.

α-Glucosidase inhibition assay

α-glucosidase enzyme (from *Saccharomyces cerevisiae*) inhibition activity was measured, using previously described method with minor modifications (3). Briefly, 5 µL of each MeOH and 80% MeOH extracts of the four seaweeds, were incubated in 96-well microplates followed by diluting with 90 µL of 0.1 mM potassium phosphate buffer (pH 6.8). After adding 20 µL of α-glucosidase enzyme (0.25 U/mL) in phosphate buffer solution, the plates were incubated in the dark condition for 10 min at 37 ºC. Afterwards, 15 µL of 2.5 mM substrate (*p*-nitrophenyl-α-D-glucopyranoside) in buffer was added into the mixture and subsequently were stored at 37 ºC for further 30 min. Finally, 80 µl (0.2 M) Na_2CO_3 was added into each well to quench the reaction and the absorbance reading (A) was measured at 405 nm using a microplate reader (Bio-Rad Model 680). The results were compared to a control solution, which involved 5µL buffer instead of the extract. In addition, a solution of acarbose in deionized water was used as the standard inhibitor. The percentage of α-glucosidase inhibitory activity was calculated as follows:

$$\% \text{ Inhibition} = (A_{control} - A_{sample}) / A_{control} \times 100$$

Where A_{sample} is the absorbance of the extract or positive standard and $A_{control}$ is the absorbance of the negative control. The IC_{50}, was defined as the concentration (µg/mL) of the sample that inhibited 50% of the enzyme activity, which were evaluated using a serial dilution of the samples, from 8 to 120 µg/mL in MeOH and 80% MeOH, using Curve Expert 1.4 software (three replicates).

Kinetic of inhibition patterns on α-glucosidase

The kinetic of the enzyme was carried out using the extracts of *S. boveanum* against α-glucosidase inhibition. Appendix 4 shows the Lineweaver-Burk plot of α-glucosidase inhibitory activity of the 80% MeOH extract of *S. boveanum* at 0, 1 and 2 mg/mL stock solution, with various substrate concentrations of PNPG (1–10 mM) (4, 5). Statistical analysis results using one-way analysis of variance (ANOVA) followed by Tukey post-hoc test, showed that 1/V $(\Delta OD/min)^{-1}$ for 1 and 2 mg/mL stock solution of the alga in 1/S = 1, 0.5 and 0.33 mM^{-1}, were significantly different (p value < 0.05) from that of the control group (no inhibitor). Data analysis revealed that, the increase in the extract's concentration has not affected the V_{max} and remained at about 0.09 mM/min, while K_m was increased significantly by 3.0, 5.4 and 24.9 mM, respectively (Appendix 4). These results indicated that the extract inhibits α-glucosidase by competitive manner.

Experimental animals

We purchased about thirty healthy adult male Sprague–Dawley rats (180-220 g) from the Center of Comparative and Experimental Medicine, Shiraz University of Medical Sciences (Shiraz, Iran). The rats were fed on a standard laboratory diet and housed at room temperature for about 12 h light and dark cycles. The local Ethics Committee of Shiraz University of Medical Sciences approved the study protocols (No. IR.SUMS.REC.1399.279).

Induction of diabetes

One week after adaptation, diabetes was induced by a single intraperitoneal injection of 50 mg/Kg of streptozotocin (STZ; Sigma-Aldrich, UK). The rats were fasted for 10 h prior to the drug administration. STZ was dissolved in sodium citrate buffer solution (pH 4.5) and instantly injected to prevent degradation. A blood sample was collected from the tail vein, five days after

STZ injection. Rats with blood glucose over 300 mg/dL were considered as diabetics (6). The Accu- Chek blood glucose test meter (Roche Diagnostic, Germany) was used for plasma glucose concentration checking.

Sucrose tolerance test

After an overnight fasting, diabetic animals were divided into three groups: 1. Diabetic controls, which received deionized water. 2. Positive controls, diabetic rats which were treated with acarbose (30 mg/Kg) as a standard antidiabetic drug (7). 3. Diabetic rats treated with 80% MeOH extract of *S. boveanum* (30 mg/Kg, the same dosage as acarbose, for the better comparison). Half an hour after the above-mentioned oral treatments, sucrose solution (2 g/Kg) was administrated orally. Plasma glucose levels were taken from the tail vein at 0, 30, 60, 90, 120 min after sucrose administration and compared among groups.

Statistical analysis

The data were displayed as mean ± standard error (SE). The statistical analysis was carried out using Excel 2016 software and inhibitory concentration (IC_{50}) values were calculated using CurveExpert 1.4 software. SPSS software (Ver. 16) was another software which was used to evaluate the results by one-way analysis of variance (ANOVA) followed by post-hoc Dunnett's multiple range test. *P*-values less than 0.05 is considered significant.

References

(1) Wagner H, Bladt S. Plant drug analysis: a thin layer chromatography atlas: Springer Science & Business Media; 1996.

(2) Firuzi O, Miri R, Asadollahi M, Eslami S, Jassbi AR. Cytotoxic, antioxidant and antimicrobial activities and phenolic contents of eleven *Salvia* species from Iran. *Iran J Pharm Res*. 2013;12(4):801-10. [PubMed: 24523760]. [PubMed Central: PMC3920696].

(3) Park SR, Kim JH, Jang HD, Yang SY, Kim YH. Inhibitory activity of minor phlorotannins from *Ecklonia cava* on α-glucosidase. *Food Chem*. 2018;257:128-34. doi: 10.1016/j.foodchem.2018.03.013. [PubMed: 29622188].

(4) Ali M, Kim DH, Seong SH, Kim H-R, Jung HA, Choi JS. α-Glucosidase and protein tyrosine phosphatase 1B inhibitory activity of plastoquinones from marine brown alga *Sargassum serratifolium*. *Mar drugs*. 2017;15(12):368. doi: 10.3390/md15120368. [PubMed: 29194348]. [PubMed Central: PMC5742828].

(5) Lineweaver H, Burk D. The determination of enzyme dissociation constants. *J Am Chem Soc*. 1934;56(3):658-66. doi: 10.1021/ja01318a036.

(6) Leiter EH. Selecting the “right” mouse model for metabolic syndrome and type 2 diabetes research. In: Stocker C, editor. Type 2 Diabetes, Methods in Molecular Biology. 560: Springer; 2009. p. 1-17. doi: 0.1007/978-1-59745-448-3_1. [PubMed: 29194348].

(7) Jia R-B, Li Z-R, Wu J, Ou Z-R, Zhu Q, Sun B, et al. Physicochemical properties of polysaccharide fractions from Sargassum fusiforme and their hypoglycemic and hypolipidemic activities in type 2 diabetic rats. *International journal of biological macromolecules*. 2020;147:428-38.

Appendix 1. Species, voucher numbers, and collection details of all the investigated algae from the Persian Gulf

Species	Source	Latitude and longitude	Voucher number
Sargassum boveanum (J. Agardh)	Shoghab Park, Bushehr	N 28° 54′ 55" E 50° 48′ 49"	Pc-93-6-2-2.1
P. perforata (K. W. Nam)	Bandar Lengeh, Hormozgan	N 26° 33′ 30" E 54° 53′ 35"	Pc-93-6-6-1.1
Polycladia myrica (Draima, Ballesteros, F. Rousseau & T. Thibaut)	Bandar Lengeh, Hormozgan	N 26° 33′ 30" E 54° 53′ 35"	Pc-93-6-12-1.3
Padina antillarum (kützing) Piccone	Bandar-e-Parsian , Hormozgan	N 27° 06′ 40" E 53° 02′ 26"	Pc-93-7-4-2.1

Appendix 2. The algal extraction parameters.

**Fresh Algae***	Extraction solvent	Solvent volume (L)	Extract weight (g)	Yield (%)
Sargassum boveanum (500 g)	MeOH	5	1.71	0.34
	80% MeOH	5	10.56	2.11
Palisada perforata (350 g)	MeOH	3	2.41	0.68
	80% MeOH	3	8.36	2.39
Padina antillarum (420 g)	MeOH	4	4.16	0.99
	80% MeOH	4	8.9	2.12
Polycladia myrica (600 g)	MeOH	6	5.07	0.84
	80% MeOH	6	10.29	1.71

*Fresh algae was used for extractions.

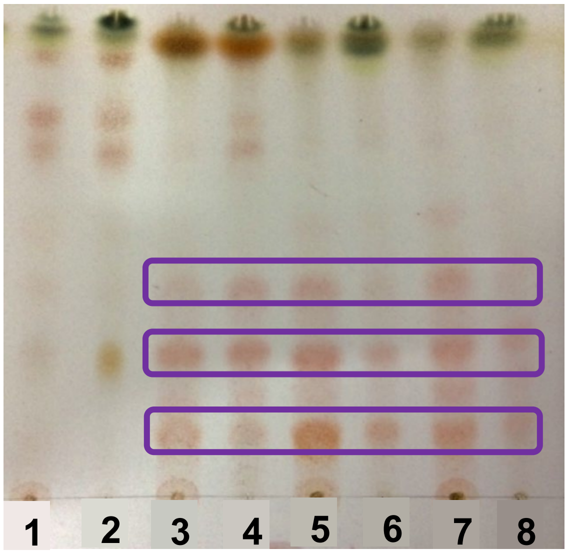

Appendix 3. Spots in daylight after spray with thymol reagent using a TLC plate and Ethyl acetate/MeOH/Formic acid (7/3.5/1) mobile phase. Spots are 80% MeOH (1),and MeOH (2) extracts of *S. boveanum*, 80% MeOH (3) and MeOH (4) extracts of *P. perforata*, 80% MeOH (5) and MeOH (6) extracts of *P. antillarum*, 80% MeOH (7) and MeOH (8) extracts of *P. myrica.*

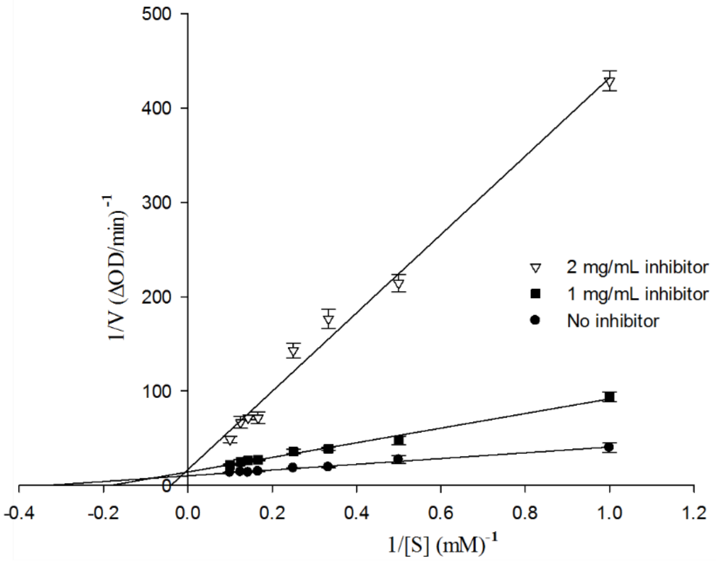

Appendix 4. The kinetics of α-glucosidase inhibition of the extract of 80% MeOH *S. boveanum* using Lineweaver-Burk plot analysis. [α-Glucosidase] = 0.25 U/mL; in 0, 1 and 2 mg/mL inhibitor. pH 6.8, at room temperature. Each value is expressed as mean ± SE.
